# Supplementary figures and images for: Hyperoside alleviates doxorubicin-induced myocardial cells apoptosis by inhibiting the apoptosis signal-regulating kinase 1/p38 pathway
Source: PeerJ. 2023 May 18;11:e15315. doi: 10.7717/peerj.15315 (PMC10200097; doi:10.7717/peerj.15315)

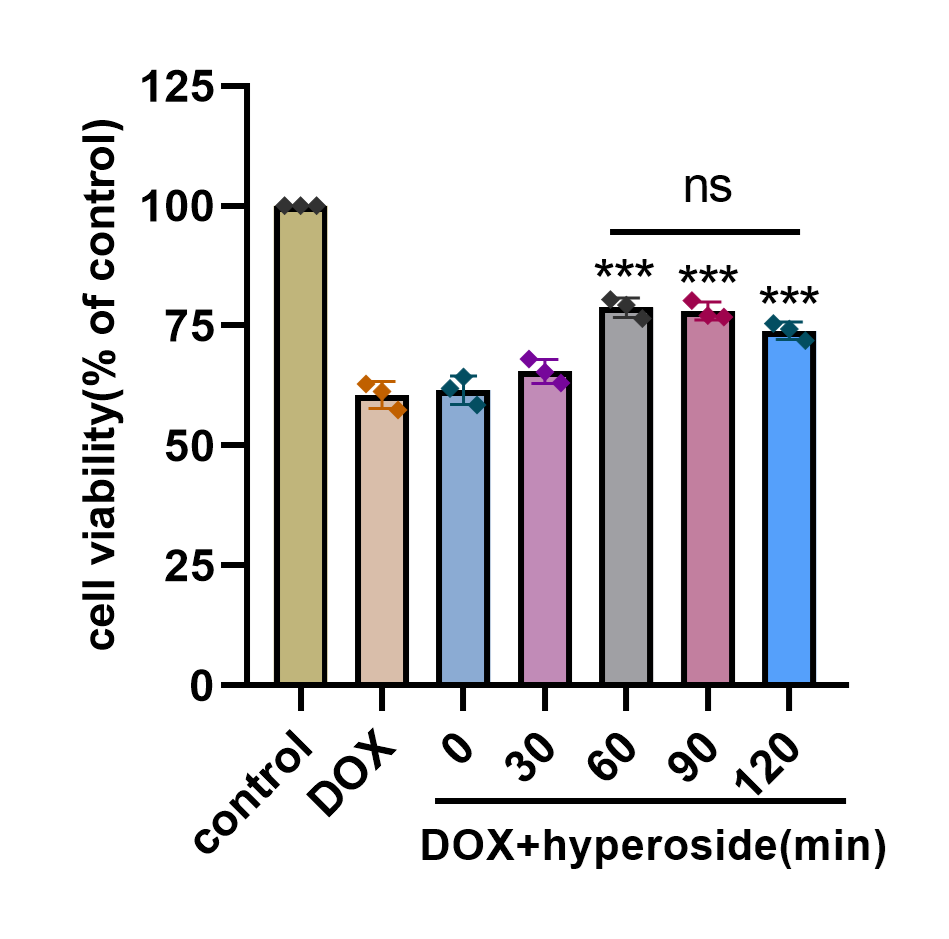

Supplement: Supplemental Information 1 — Effects of hyperoside on the survival rate of HL-1 cells (n = 3). *** P < 0.001 compared with the DOX group. “ns”: P was no significance. [file peerj-11-15315-s001.png]

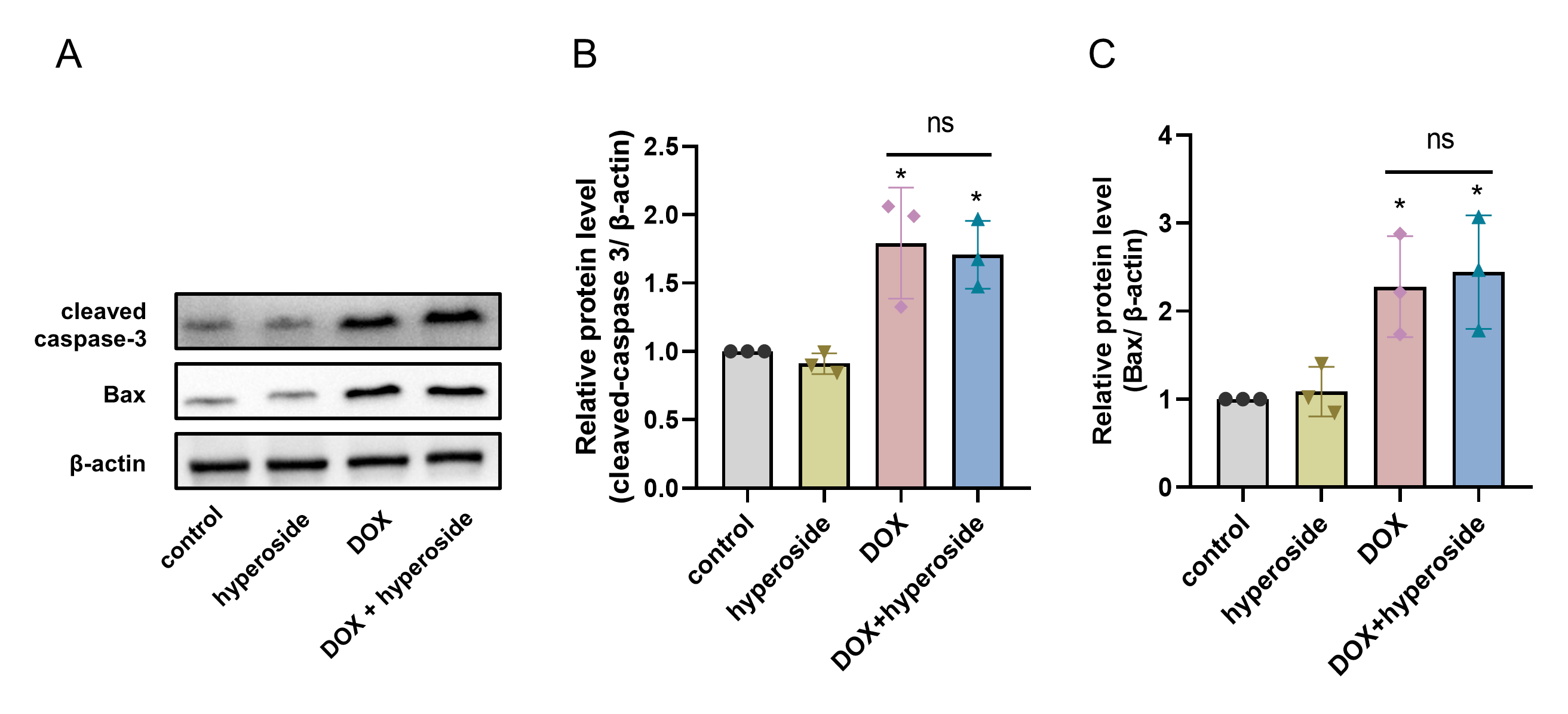

Supplement: Supplemental Information 2 — (A) The expression of cleaved caspase-3 and Bax in HL-1 cells assessed by western blot analysis. β-actin was used as the control. (B) Quantitative analysis of Bax depicted by bar graph (n = 3). (C) Quantitative analysis of cleaved caspase-3 depicted by bar graph (n = 3). * P < 0.05 compared with the control group. “ns”: P was no significance. [file peerj-11-15315-s002.png]
